# THE TRAJECTORY OF FAMILY FUNCTION OF STROKE SURVIVORS AND ITS IMPACT ON COGNITIVE IMPAIRMENT: A LONGITUDINAL STUDY

**DOI:** 10.1093/geroni/igac059.071

**Published:** 2022-12-20

**Authors:** Juan Li, Zhijian Liu, Fanfan Li, Jing Wang, Bei Wu

**Affiliations:** Huashan Hospital affiliated to Fudan University, Shanghai, Shanghai, China (People's Republic); Naval Military Medical University, Shanghai, Shanghai, China (People's Republic); Naval Military Medical University, Shanghai, Shanghai, China (People's Republic); University of North Carolina at Chapel Hill, Chapel Hill, North Carolina, United States; New York University, New York, New York, United States

## Abstract

This study examined the trajectory of family function of Chinese stroke survivors and its impact on cognitive impairment.We conducted a prospective longitudinal study and assessed family function of 170 stroke survivors and their family caregivers by Family Assessment Device(FAD) during acute stage and at 3, 6 months after onset via face-to-face follow-ups interview in Nanjing, China from January to October, 2020.Group-based Trajectory Model was applied to analyze trajectories of family function. Family function deteriorated with time. Four patterns of FAD trajectory (A: dysfunction-deterioration-improvement; B: severe dysfunction-stable; C: healthy function-rapid deterioration; D: approximate healthy function-stable) were identified. Healthy and stable family function was associated with better cognition and quality of life (β=-0.216,p<0.05, β=-0.159, p<0.05, respectively). Future studies are needed to further explore the reason why family function deteriorated and the linkage between family function and stroke survivors’ outcomes.

